# Design, Synthesis and Anticancer Evaluation of Novel Quinazoline-Sulfonamide Hybrids

**DOI:** 10.3390/molecules21020189

**Published:** 2016-02-04

**Authors:** Mostafa M. Ghorab, Mansour S. Alsaid, Mohammed S. Al-Dosari, Marwa G. El-Gazzar, Mohammad K. Parvez

**Affiliations:** 1 Department of Pharmacognosy, College of Pharmacy, King Saud University, P.O. Box 2457, Riyadh 11451, Saudi Arabia; msalsaid@ksu.edu.sa (M.S.A.); msdosari@yahoo.com (M.S.A.-D.); khalid_parvez@yahoo.com (M.K.P.); 2 Department of Drug Radiation Research, National Center for Radiation Research and Technology, Atomic Energy Authority, P.O. Box 29, Nasr City, Cairo 11371, Egypt; marwagalalgazzar@yahoo.com

**Keywords:** synthesis, quinazoline, sulfonamides, anticancer activity

## Abstract

By combining the structural features of quinazoline and sulfonamides, novel hybrid compounds **2**–**21** were synthesized using a simple and convenient method. Evaluation of these compounds against different cell lines identified compounds **7** and **17** as most active anticancer agents as they showed effectiveness on the four tested cell lines. The anticancer screening results of the tested compounds provides an encouraging framework that could lead to the development of potent new anticancer agents.

## 1. Introduction

**Figure 1:**
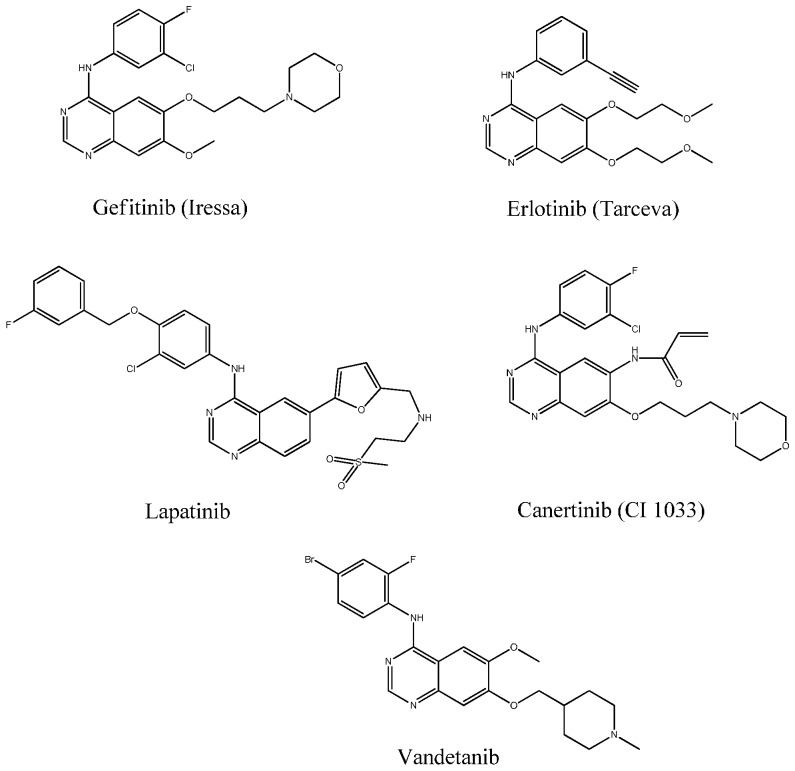
EGFR-tyrosine kinase inhibitors.

Cancer continues to be a leading health problem in developed as well as developing countries. It has become the number one killer due to various worldwide factors [1,2,3,4,5]. This enormous incidence of cancer has increased the urgency of the search for the latest, safer and efficacious anticancer agents, aiming at the prevention or the cure of this illness [6,7,8]. In the efforts to identify various chemical substances which may serve as leads for designing novel anticancer agents, nitrogen- and sulfur- containing heterocycles are of particular interest [9,10,11]. Quinazoline and sulfonamide moieties have been identified as classes of cancer chemotherapeutic agents with significant therapeutic efficacy against solid tumors. In recent years, quinazolines, as an important pharmacophore, have emerged as a versatile template for inhibition of a diverse range of receptor tyrosine kinases [12,13,14,15,16]. The most widely studied of these is the epidermal growth factor receptor (EGFR), with the small-molecule inhibitor gefitinib being the first quinazoline derivative to be approved for the treatment of Non-Small Cell Lung Cancer [17,18,19,20,21]. Subsequent research aimed at further exploration of the SAR of this novel template has led to discovery of highly selective compounds that target EGFR such as erlotinib, lapatinib, canertinib and vandetanib [22,23,24] (Figure 1). These compounds act via competing with ATP for binding at the catalytic domain of tyrosine kinase. Later on, a great structural variety of compounds of structurally diverse classes have proved to be highly potent and selective ATP-competitive inhibitors [25,26,27,28]. Based on the good performances of quinazoline derivatives in anticancer applications, the development of novel quinazoline derivatives as anticancer drugs is a promising field. 

**Figure 2:**
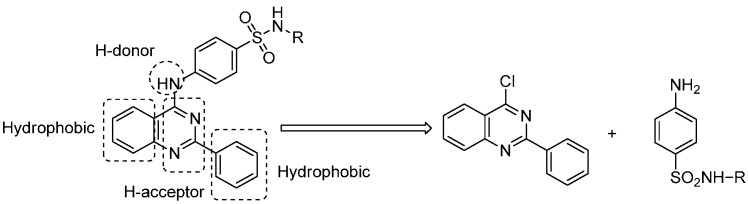
The designed quinazoline-sulfonamide hybrids.

Varied biological activities have been attributed to sulfonamide compounds, including carbonic anhydrase inhibition, antitumoral, antimalarial and antimicrobial activities [29,30,31]. In the design of new drugs, the development of hybrid molecules through the combination of different pharmacophores may lead to compounds with interesting biological profiles [32]. In view of the abovementioned knowledge about different pharmacophores and in continuation of our research programme [33,34,35,36,37,38,39,40,41,42,43,44,45], we have now synthesized quinazoline-sulfonamide hybrids to obtain a single molecular framework incorporating both moieties. These hybrid molecules consist of a planar heterocyclic ring (quinazoline) with a hydrophobic phenyl ring at position-2 as a central core that can act as a scaffold to carry a functionalized branch at position-4, in such a way to accommodate a sulfonamide moiety (Figure 2). Introduction of the benzensulfonamide amino group at position-4 will add a new hydrogen bond donor, a very much needed characteristic for the desired activity [46]. These compounds were then screened for their *in vitro* anticancer activity against various cell lines.

## 2. Results and Discussion

### 2.1. Chemistry

**Scheme 1:**
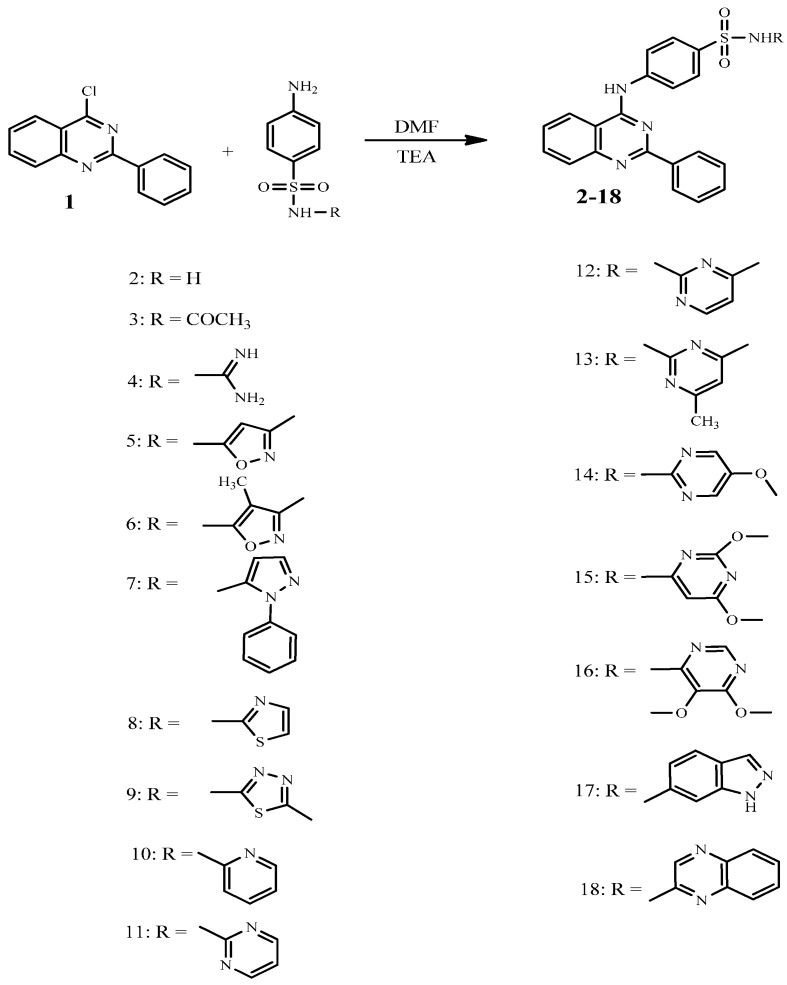
Synthetic pathways for compounds **2**–**18**.

**Scheme 2:**
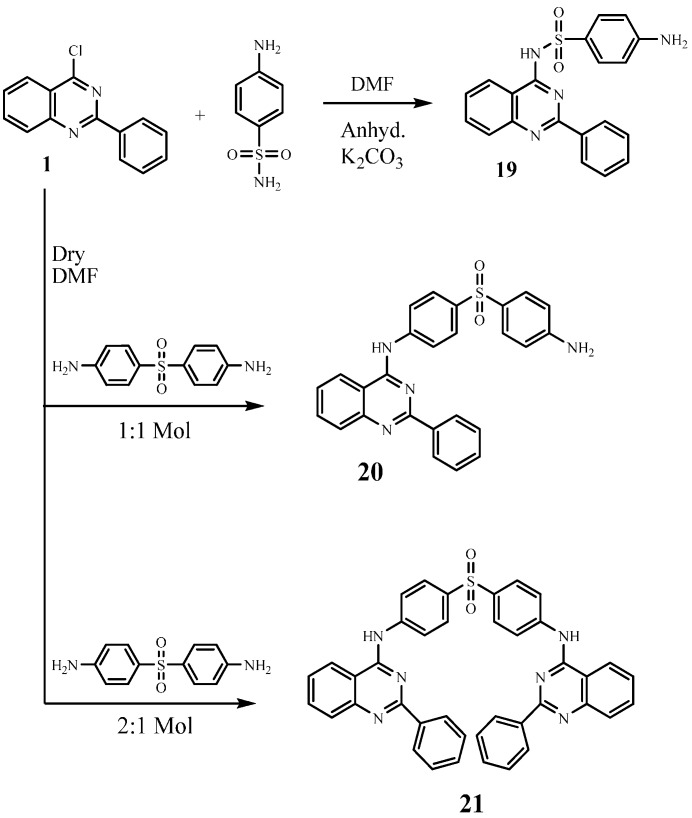
Synthetic pathways for compounds **19**–**21**.

The aim of this work was to design and synthesize novel quinazoline-sulfonamide hybrids to evaluate their anticancer activity. Thus, interaction of 4-chloro-2-phenylquinazoline (**1**) with several sulfonamides in dry *N*,*N*-dimethylformamide afforded the corresponding quinazoline-sulfonamide derivatives **2**–**18** (Scheme 1). The structures of the formed compounds were confirmed on the basis of elemental analyses and spectral data. Thus, the IR spectra of compounds **2**–**18** showed absorption bands for (NH), (CH aromatic), (CH aliphatic), (C=N) and (SO_2_) functional groups. The ^1^H-NMR spectra exhibited singlets assigned to the NH group which were exchanged upon deuteration. Also, interaction of compound **1** with sulfanilamide in dimethylformamide in the presence of anhydrous K_2_CO_3_ furnished 4-amino-*N*-(2-phenylquinazoline-4-yl)-benzenesulfonamide (**19**, Scheme 2), this reaction proceeded through salt formation of the acidic amino group of sulfonamide (SO_2_NH_2_) which further reacted with the chloro group of the quinazoline to yield compound **19**. 

The IR spectrum of compound **19** showed characteristic bands at 3412, 3209 cm^−1^ (NH, NH_2_), 3084 cm^−1^ (CH aromatic) 1635 cm^−1^ (C=N), 1375, 1134 cm^−1^ (SO_2_). The ^1^H-NMR spectrum of compound **19** exhibited signals at 7.0 ppm due to the NH_2_ group, which exchangeable with D_2_O, and a singlet at 11.9 ppm assigned to the NH group which was exchangeable with D_2_O. The mass spectrum of compound **19** revealed a molecular ion peak *m*/*z* at of 376[M^+^] (11.64). In addition, by interaction of compound **1** with dapsone in 1:1 molar ratio, *N*-(4-(4-aminophenylsulfonyl)phenyl)-2-phenylquinazolin-4-amine (**20**) was formed, while, an additional mole of 2-phenylquinazolin-4-amine was introduced to give the bis-compound **21** under the same reaction conditions but using a 2:1 molar ratio. The structures of compounds **20** and **21** were confirmed on the basis of elemental analyses, IR, ^1^H-NMR, ^13^C-NMR and mass spectral data. The IR spectrum of compound **20** revealed characteristic bands at 3375, 3257, (NH, NH_2_), 1664 (C=O), 1618 (C=N), 1389, 1140 (SO_2_). The ^1^H-NMR spectrum of compound **20** exhibited signals at 5.9 ppm due to a NH_2_ group, which was exchangeable with D_2_O, and a singlet at 10.1 ppm assigned to a NH group which was exchangeable with D_2_O. The mass spectrum of compound **20** revealed a molecular ion peak at *m*/*z* 453 [M^+^] (13.72). The IR spectrum of **21** showed characteristic bands at 3367 (2NH), 1622 (2C=N), 1375, 1181 (SO_2_). The ^1^H-NMR spectrum of **21** revealed signals at 10.1 ppm corresponding to two NH groups which were exchangeable with D_2_O. The mass spectrum of compound **21** showed a molecular ion peak at *m*/*z* 657 [M^+^] (32.18); this increase in the mass demonstrated the introduction of the second mole of dapsone.

### 2.2. In-Vitro Anticancer Evaluation

The synthesized compounds were evaluated for their *in vitro* anticancer activity against human lung cancer cell line (A549), cervical (HeLa) cancer cell line, colorectal cell line (LoVo) and breast cancer cell line (MDA-MB-231) using doxorubicin as reference drug. The relationship between surviving fraction and drug concentration was plotted to obtain the survival curves of the cancer cell lines. The response parameter calculated was the IC_50_ value, which corresponds to the concentration required for 50% inhibition of cell viability. The results are presented in Table 1, where all compounds exhibit moderate activity compared to doxorubicin as positive control. 

**Table 1:** *In vitro* anticancer screening of the synthesized compounds against four cell lines. Data are expressed as IC_50_ (µM) ± SD (*n* = 3).

Cpd. No.	A549 (Lung Cancer Cells)	HeLa (Cervical)	LoVo (Colorectal Cancer Cells)	MDA-MB-231 (Breast Cancer Cells)
**2**	134.9 ± 0.40	NA	94.9 ± 0.78	58.2 ± 1.76
**3**	113.5 ± 1.10	221.1 ± 1.22	80.5 ± 0.87	51.4 ± 1.32
**4**	129.4 ± 0.71	187.7 ± 1.10	61.7 ± 1.31	36.4 ± 0.34
**5**	NA	NA	212.8 ± 0.78	NA
**6**	NA	NA	217.0 ± 1.11	NA
**7**	77.8 ± 0.54	91.5 ± 0.41	96.5 ± 0.34	77.9 ± 0.36
**8**	130.4 ± 0.67	284.6 ± 1.03	160.1 ± 0.90	97.4 ± 1.40
**9**	NA	NA	182.5 ± 0.33	NA
**10**	NA	NA	125.4 ± 0.88	154.1 ± 1.12
**11**	NA	NA	54.2 ± 0.92	NA
**12**	NA	NA	58.6 ±0.50	NA
**13**	NA	NA	112.9 ± 0.35	NA
**14**	NA	NA	101.7 ± 0.67	93.9 ± 0.45
**15**	NA	NA	61.5 ± 0.01	NA
**16**	NA	NA	65.5 ± 1.65	NA
**17**	161.6 ± 0.78	87.6 ± 1.00	97.3 ± 0.23	42.8 ± 1.09
**18**	NA	276.0 ± 1.01	132.0 ± 1.04	NA
**19**	NA	NA	146.4 ± 1.34	NA
**20**	NA	251.6 ± 0.98	72.5 ± 0.26	NA
**21**	NA	189.8 ± 1.12	73.1 ± 1.54	NA
Doxorubicin	283.5 ± 0.01	120.7 ± 0.09	374.4 ± 1.00	26.5 ± 0.54

NA = Not Active.

In the case of the human lung cancer cell line (A549) compounds **2**, **3**, **4**, **7**, **8**, and **17** were the most potent, with IC_50_ values ranging from 77.8–161.6 µM, lower than the reference. On the other hand, the tested compounds showed low activity on the HeLa cell line where he most potent were the sulfonamide derivatives **7** and **17** (IC_50_ = 91.5 and 87.6 µM, respectively). In case of the colorectal cell line LoVo, all compounds showed excellent activity and were found to be more active than doxorubicin. Moderate activity was observed for the synthesized compounds on the MDA-MB-231 breast cancer cell line, where the most potent candidates were compounds **2**–**4**, **7**, **8**, **10**, **14** and **17**, which all showed lower activity than the reference drug doxorubicin. Generally, the colorectal (LoVo) and breast (MDA-MB-231) cancer cell lines were the most sensitive to the synthesized compounds. With regard to broad spectrum anticancer activity, close examination of the data presented in Table 1, reveals that compounds **7** and **17** were the most active, showing effectiveness toward the four cell lines.

## 3. Materials and Methods

### 3.1. General Information

All analyses were done at the Research Center, King Saud University (Riyadh, Saudi Arabia). Melting points (uncorrected) were determined in open capillaries on a Gallenkamp melting point apparatus (Sanyo Gallenkamp, Southborough, UK). Precoated silica gel plates (Kieselgel 0.25 mm, 60 F254, Merck, Darmstadt, Germany) were used for thin layer chromatography. A developing solvent system of 4:1 chloroform/methanol was used and the spots were detected by ultraviolet light. IR spectra (KBr discs) were recorded using an FT-IR spectrometer (Perkin Elmer, Waltham, MA, USA). ^1^H-NMR spectra were scanned on NMR spectrometer (Bruker AXS Inc., Flawil, Switzerland), operating at 500 MHz for ^1^H- and 125.76 MHz for ^13^C. Chemical shifts are expressed in δ-values (ppm) relative to TMS as an internal standard, using DMSO-*d*_6_ as a solvent. Mass spectra were recorded on a 600 GC/MS (Clarus, Middletown, CT, USA) and TQ 320 GC/MS/MS mass spectrometers (Varian, West Sussex, UK). Elemental analyses were done on a model 2400 CHNSO analyser (Perkin Elmer, Waltham, MA, USA). All the values were within ± 0.4% of the theoretical values. All reagents used were of AR grade. The starting material 4-chloro-2-phenylquinazoline was purchased from Sigma (St. Louis, MO, USA) and was directly used for the preparation of target compounds. Spectroscopic data of the synthesized compounds can be accessed as supplementary materials.

### 3.2. General Procedure for the Synthesis of Sulfonamide Derivatives **2**–**18**

A mixture of 4-chloro-2-phenylquinazoline (**1**, 2.42 g, 0.01 mol) and sulfonamides (0.012 mol) in dry dimethylformamide (10 mL) was refluxed for 22 h., then left to cool. The solid product formed upon pouring onto ice/water was collected by filtration and recrystallized from ethanol-dimethylformamide to give **2**–**18**, respectively.

*4-(2-Phenylquinazolin-4-ylamino)benzenesulfonamide* (**2**). Yield, 89%; m.p. 209.3 °C. IR (KBr, cm^−1^): 3196, 3169, 3136 (NH, NH_2_), 3061 (CH arom.), 1670, 1602 (2C=N), 1394, 1190 (SO_2_).^1^H-NMR (DMSO-*d*_6_): 7.4–8.5 (m, 15H, Ar-H + SO_2_NH_2_), 9.7 (s, 1H, NH exchangeable with D_2_O). ^13^C-NMR (DMSO-*d*_6_): 114.4 (2), 121.4, 125.6, 126.3 (2), 127.0, 127.9, 128.3 (2), 128.8 (2), 129.2, 130.4, 131.1, 133.0, 139.1, 149.2, 152.7, 160.2. MS *m*/*z* (%): 376 (M^+^) (23.42), 74 (100). Anal. Calcd. For C_20_H_16_N_4_O_2_S (376): C, 63.81; H, 4. 28; N, 14.88. Found: C, 63.53; H, 4.50; N, 14.49.

*N-(4-(2-Phenylquinazolin-4-ylamino)phenylsulfonyl)acetamide* (**3**). Yield, 91%; m.p. 243.5 °C. IR (KBr, cm^−1^): 3412, 3269 (NH), 3100 (CH arom.), 2956, 2843 (CH aliph.), 1667 (C=O), 1602, 1571 (2C=N), 1344, 1189 (SO_2_). ^1^H-NMR (DMSO-*d*_6_): 1.9 (s, 3H, COCH3), 7.3–8.7 (m, 14H, Ar-H + SO_2_NH), 12.5 (s, 1H, NH, exchangeable with D_2_O). ^13^C-NMR (DMSO-*d*_6_): 23.6, 121.4 (2), 126.3, 126.8, 127.0 (2), 127.8 (2), 128.2 (2), 129.0, 129.1 (2), 131.8 (2), 133.1, 135.0, 149.0 (2), 152.8, 162.7. MS *m*/*z* (%): 418 (M^+^) (41.31), 122 (100). Anal. Calcd. For C_22_H_18_N_4_O_3_S (418): C, 63.14; H, 4. 34; N, 13.39. Found: C, 63.43; H, 4.10; N, 13.69.

*N-Carbamimidoyl-4-(2-phenylquinazolin-4-ylamino)benzenesulfonamide* (**4**). Yield, 78%; m.p. 314.4 °C. IR (KBr, cm^−1^): 3425, 3329, 3186 (NH, NH_2_), 3100 (CH arom.), 2928,2868 (CH aliph.), 1669, 1618, 1601 (C=N), 1397,1169 (SO_2_).^1^H-NMR (DMSO-*d*_6_): 6.7 (s, 2H, NH_2_, exchangeable with D_2_O), 7.1, 8.4 (m, 14H, Ar-H + SO_2_NH), 8.6 (s, 1H, NH imino, exchangeable with D_2_O) ,10.1 (s, 1H,NH, exchangeable with D_2_O). ^13^C-NMR (DMSO-*d*_6_): 114.4 (2), 121.4, 123.6, 126.3 (2), 127.0, 128.2, 128.4 (2), 129.0 (2), 131.0, 131.8, 133.1, 134.1, 139.5, 142.3, 158.2, 159.3, 162.7.MS *m*/*z* (%): 418 (M^+^) (25.4), 76 (100). Anal. Calcd. For C_21_H_18_N_6_O_2_S (418): C, 60.27; H, 4. 34; N, 20.08. Found: C, 60.55; H, 4.09; N, 20.31.

*N-(3-Methylisoxazol-5-yl)-4-(2-phenylquinazolin-4-ylamino)benzenesulfonamide* (**5**). Yield, 83%; m.p. 133.4 °C. IR (KBr, cm^−1^): 3323, 3196 (NH), 3061 (CH arom.), 2927, 2871 (CH aliph.), 1670, 1622, 1600 (C=N), 1357,1143 (SO_2_).^1^H-NMR (DMSO-*d*_6_): 2.2 (s, 3H, CH_3_), 6.8 (s, 1H, CH isoxazole), 7.1–8.6 (m, 13H, Ar-H),10.2 (s, 1H, SO_2_NH, exchangeable with D_2_O),12.5 (s,1H,NH exchangeable with D_2_O). ^13^C-NMR (DMSO-*d*_6_):10.9, 98.3, 114.5 (2), 114.7, 123.5, 126.3 (2), 126.6, 127.0, 128.2 (2), 128.9, 130.5 (2), 131.8, 132.7, 135.0, 141.6, 149.2, 158.1, 159.3, 162.7, 163.2. MS *m*/*z* (%): 458 (M^+^) (24.54), 81 (100). Anal. Calcd. For C_24_H_19_N_5_O_3_S (458): C, 63.01; H, 4. 19; N, 15.31. Found: C, 63.29; H, 4.45; N, 15.61.

*N-(3,4-Dimethylisoxazol-5-yl)-4-(2-phenylquinazolin-4-yl-amino)benzenesulfonamide* (**6**). Yield, 77%; m.p. 114.0 °C. IR (KBr, cm^−1^): 3323, 3196 (NH), 3061 (CH arom.), 2927,2819 (CH aliph.), 1670, 1624 (C=N), 1373,1143 (SO_2_).^1^ H-NMR (DMSO-*d*_6_):2.6, 2.7 (2s, 6H, 2CH_3_), 7.3, 8.6 (m, 13H, Ar-H), 10.1 (s, 1H,SO_2_NH, exchangeable with D_2_O), 12.5 (s, 1H, NH, exchangeable with D_2_O). ^13^C-NMR (DMSO-*d*_6_): 6.8, 10.8, 101.8, 114.5 (2), 114.7, 124.7, 126.6 (2), 127.2, 128.2, 128.6(2), 128.9, 129.0 (2), 130.5, 131.5, 132.7, 143.0, 149.2, 158.1, 159.3, 162.7, 163.2.MS *m*/*z* (%): 472 (M^+^) (4.7), 65 (100). Anal.Calcd. For C_25_H_21_N_5_O_3_S (472): C, 63.68; H, 4. 49; N, 14.85. Found: C, 63.37; H, 4.27; N, 14.59.

*N-(1-Phenyl-1H-pyrazol-5-yl)-4-(2-phenylquinazolin-4-ylamino)benzenesulfonamide* (**7**). Yield, 89%; m.p. 232.6 °C. IR (KBr, cm^−1^): 3196, 3134 (NH), 3064 (CH arom.), 1670, 1602 (C=N), 1340, 1190 (SO_2_).^1^H-NMR (DMSO-*d*_6_): 7.4–8.5 (m, 20H, Ar-H), 12.5 (s, 1H, SO_2_NH + NH, exchangeable with D_2_O). ^13^C-NMR (DMSO-*d*_6_): 97.9, 115.2 (2), 121.4, 126.3 (2), 127.0 (2), 127.9, 128.2 (2), 128.3, 128.4, 128.7 (2), 128.9 (2), 129.0, 131.8 (2), 131.8, 133.2 (2), 135.0 (2), 149.2, 152.7, 162.7 (2). MS *m*/*z* (%): 519 (M^+^) (4.43), 103 (100). Anal.Calcd. For C_29_H_22_N_6_O_2_S (519): C, 67.17; H, 4. 28; N, 16.21. Found: C, 67.48; H, 4.52; N, 16.50.

*4-(2-Phenylquinazolin-4-ylamino)-N-(thiazol-2-yl)benzenesulfonamide* (**8**). Yield, 79%; m.p. 146.7 °C. IR (KBr, cm^−1^): 3487, 3381 (NH), 3084 (CH arom.), 1622, 1599 (C=N), 1358, 1178 (SO_2_). ^1^H-NMR (DMSO-*d*_6_): 6.8–8.6 (m, 15H, Ar-H), 10.1 (s,1H,SO_2_NH, exchangeable with D_2_O), 12.7 (s, 1H,NH, exchangeable with D_2_O).^13^C-NMR (DMSO-*d*_6_): 108.5, 114.5 (2), 121.6, 124.9, 126.3 (2), 126.7, 127.0, 128.2 (2), 128.8, 130.9 (2), 131.8, 133.1, 133.9, 136.8, 143.2, 151.0, 158.1, 162.7, 169.1. MS *m*/*z* (%): 460 (M^+^) (9.59), 93 (100). Anal. Calcd. For C_23_H_17_N_5_O_2_S_2_ (460): C, 60.11; H, 3.73; N, 15.24. Found: C, 60.43; H, 3.44; N, 15.50.

*N-(5-Methyl-1,3,4-thiadiazol-2-yl)-4-(2-phenylquinazolin-4-ylamino)benzenesulfonamide* (**9**). Yield, 80%; m.p. 188.9 °C. IR (KBr, cm^−1^): 3412, 3349 (NH), 3061 (CH arom.), 2923, 2859 (CH aliph.), 1622, 1600 (C=N), 1358,1184 (SO_2_).^1^H-NMR (DMSO-*d*_6_): 2.4 (s, 3H**,** CH_3_), 7.3–8.6 (m, 13H, Ar-H), 10.1 (s,1H,SO_2_NH, exchangeable with D_2_O), 12.9 (s, 1H, NH, exchangeable with D_2_O). ^13^C-NMR (DMSO-*d*_6_): 16.5, 114.5 (2), 114.6, 124.9 (2), 126.3, 126.7, 127.9, 128.4, 129.0 (2), 130.7 (2), 131.8, 133.0, 134.0, 138.5, 143.6, 149.2, 152.7, 158.1, 168.2. MS *m*/*z* (%): 474 (M^+^) (20.8), 163 (100). Anal. Calcd. For C_23_H_18_N_6_O_2_S_2_ (474): C, 58.51; H, 3.82; N, 17.71. Found: C, 58.19; H, 3.58; N, 17.49.

*4-(2-Phenylquinazolin-4-ylamino)-N-(pyridine-2-yl)benzenesulfonamide* (**10**). Yield, 91%; m.p. 232.1 °C. IR (KBr, cm^−1^): 3365, 3209 (NH), 3067 (CH arom.), 1635, 1600 (C=N), 1355, 1134 (SO_2_). ^1^H-NMR (DMSO-*d*_6_): 6.9–8.5 (m, 17H, Ar-H), 10.3 (s, 1H, SO_2_NH, exchangeable with D_2_O), 11.8 (s, 1H, NH, exchangeable with D_2_O). ^13^C-NMR (DMSO-*d*_6_): 114.0, 114.5 (2), 116.4, 121.4, 123.5, 126.4 (2), 127.0, 127.9, 128.3 (2), 129.0, 130.8 (2), 131.8, 132.7, 133.3, 135.9, 140.5, 149.2, 151.0, 153.4, 159.3, 162.7. MS *m*/*z* (%): 454 (M^+^) (28.2), 79 (100). Anal.Calcd. For C_25_H_19_N_5_O_2_S (454): C, 66.21; H, 4. 22; N, 15.24. Found: C, 66.43; H, 4.52; N, 15.55.

*4-(2-Phenylquinazolin-4-ylamino)-N-(pyrimidin-2-yl)benzenesulfonamide* (**11**). Yield, 85%; m.p. 251.9 °C. IR (KBr, cm^−1^): 3167, 3129 (NH), 3084 (CH arom.), 1635 (C=O), 1683, 1600 (C=N), 1392, 1159 (SO_2_). ^1^H-NMR (DMSO-*d*_6_): 6.9–8.5 (m, 16H, Ar-H), 10.1 (s, 1H, SO_2_NH, exchangeable with D_2_O), 12.5 (s, 1H, NH exchangeable with D_2_O). ^13^C-NMR (DMSO-*d*_6_): 114.5, 114.7 (2), 116.2, 124.7, 126.7 (2), 127.0, 128.2, 129.7 (2), 130.5, 130.9 (2), 131.8, 132.7, 133.1, 144.0, 149.2, 158.1 (2), 159.3, 162.7, 163.2. MS *m*/*z* (%): 455 (M^+^) (29.0), 158 (100). Anal. Calcd. For C_24_H_18_N_6_O_2_S (455): C, 63.42; H, 3.99; N, 18.49. Found: C, 63.14; H, 4.32; N, 18.12.

*N-(4-Methylpyrimidin-2-yl)-4-(2-phenylquinazolin-4-ylamino)benzenesulfonamide* (**12**). Yield, 78%; m.p. 261.1 °C. IR (KBr, cm^−1^): 3386, 3330 (NH), 3034 (CH arom.), 2962, 2870 (CH aliph.), 1624, 1599 (C=N), 1356,1147 (SO_2_).^1^H-NMR (DMSO-*d*_6_): 2.3 (s, 3H, CH_3_), 6.9–8.5 (m, 15H, Ar-H), 10.1 (s, 1H, SO_2_NH, exchangeable with D_2_O), 11.7 (s, 1H,NH, exchangeable with D_2_O). ^13^C-NMR (DMSO-*d*_6_):23.7, 112.5, 114.5 (2), 115.3, 124.7, 126.5 (2), 127.0, 128.2, 128.4 (2), 129.0, 130.5 (2), 131.8, 132.8, 133.1, 143.9, 149.2, 152.7, 157.1, 162.7, 163.1, 168.7. MS *m*/*z* (%): 469 (M^+^) (4.36), 171 (100). Anal. Calcd. For C_25_H_20_N_6_O_2_S (469): C, 64.09; H, 4.30; N, 17.94. Found: C, 64.30; H, 4.59; N, 17.68.

*N-(4,6-Dimethylpyrimidin-2-yl)-4-(2-phenylquinazolin-4-ylamino)benzenesulfonamide* (**13**). Yield, 90%; m.p. 232.8 °C. IR (KBr, cm^−1^): 3365, 3196 (NH), 3064 (CH arom.), 2954, 2861 (CH aliph.), 1670, 1618, 1597 (C=N), 1355,1155 (SO_2_).^1^H-NMR (DMSO-*d*_6_): 2.3 (s, 6H, 2CH_3_), 6.7 (s, 1H CH pyrimidine), 7.1–8.6 (m, 13H, Ar-H), 10.0 (s, 1H, SO_2_NH, exchangeable with D_2_O), 12.5 (s,1H,NH, exchangeable with D_2_O). ^13^C-NMR (DMSO-*d*_6_): 24.3 (2), 114.5, 114.7 (2), 121.0, 124.8, 126.7 (2), 127.0, 127.9, 128.3, 128.7 (2), 129.5 (2), 130.3, 131.8, 133.1, 138.3, 149.2, 151.0, 159.3 (2), 162.7, 163.2. MS *m*/*z* (%): 483 (M^+^) (28.71), 109 (100). Anal. Calcd. For C_26_H_22_N_6_O_2_S (483): C, 64.71; H, 4. 60; N, 17.42. Found: C, 64.45; H, 4.29; N, 17.70.

*N-(5-Methoxypyrimidin-2-yl)-4-(2-phenylquinazolin-4-ylamino)benzenesulfonamide* (**14**). Yield, 84%; m.p. 264.6 °C. IR (KBr, cm^−1^): 3423, 3221 (NH), 3100 (CH arom.), 2979, 2865 (CH aliph.), 1664, 1618, 1600 (C=N), 1356, 1161 (SO_2_).^1^H-NMR (DMSO-*d*_6_): 3.7 (s, 3H, OCH_3_), 7.5–8.6 (m, 15H, Ar-H), 10.3 (s, 1H, SO_2_NH, exchangeable with D_2_O), 11.4 (s, 1H, NH, exchangeable with D_2_O). ^13^C-NMR (DMSO-*d*_6_): 56.7, 112.6 (2), 114.5, 126.3, 126.7, 127.9 (2), 128.3, 128.7 (2), 128.8, 129.3 (2), 130.9, 131.8, 134.6, 138.0 (2), 143.9, 145.0, 149.2, 158.1, 159.3, 162.7. MS *m*/*z* (%): 485 (M^+^) (21.87), 74 (100). Anal. Calcd. For C_25_H_20_N_6_O_3_S (485): C, 61.97; H, 4.16; N, 17.34. Found: C, 61.66; H, 4.33; N, 17.60.

*N-(2,6-Dimethoxypyrimidin-4-yl)-4-(2-phenylquinazolin-4-ylamino)benzenesulfonamide* (**15**). Yield, 87%; m.p. 367.8 °C. IR (KBr, cm^−1^): 3221, 3169 (NH), 3057 (CH arom.), 2980, 2850 (CH aliph.), 1670, 1618, 1602 (C=N), 1388,1147 (SO_2_).^1^H-NMR (DMSO-*d*_6_): 3.72, 3.79 (2s, 6H, 2OCH_3_), 6.1 (s, 1H, CH pyrimidine), 7.3–8.8 (m, 13H, Ar-H), 10.0 (s, 1H, SO_2_NH, exchangeable with D_2_O), 11.4 (s, 1H,NH, exchangeable with D_2_O). ^13^C-NMR (DMSO-*d*_6_): 55.6 (2), 84.3, 114.4 (2), 121.4, 123.6, 126.7 (2), 127.0, 127.9, 128.4 (2), 129.9, 130.9 (2), 131.8, 133.1, 135.0, 142.9, 149.1, 156.0, 159.3, 161.7, 162.6, 162.7. MS *m*/*z* (%): 515 (M^+^) (5.19), 154 (100). Anal. Calcd. For C_26_H_22_N_6_O_4_S (515): C, 60.69; H, 4.31; N, 16.33. Found: C, 60.40; H, 4.62; N, 16.03.

*N-(5,6-Dimethoxypyrimidin-4-yl)-4-(2-phenylquinazolin-4-yl-amino)benzenesulfonamide* (**16**). Yield, 79%; m.p. 139.0 °C. IR (KBr, cm^−1^): 3360, 3192 (NH), 3059 (CH arom.), 2941, 2863 (CH aliph.), 1670, 1602 (C=N), 1328, 1188 (SO_2_). ^1^H-NMR (DMSO-*d*_6_): 3.73, 3.74 (2s, 6H, 2OCH_3_), 7.2–8.2 (m, 14H, Ar-H), 8.5 (s, 1H, CH pyrimidine), 10.1 (s, 1H, SO_2_NH, exchangeable with D_2_O), 12.5 (s, 1H,NH, exchangeable with D_2_O).^13^C-NMR (DMSO-*d*_6_): 59.4 (2), 114.5 (2), 114.6, 125.0, 126.5 (2), 127.9, 128.2, 128.4 (2), 129.0, 130.2 (2), 130.6, 131.8, 133.1, 134.0, 143.9, 147.2, 152.7, 158.2, 159.3, 162.7, 162.8. MS *m*/*z* (%): 515 (M^+^) (28.12), 168 (100). Anal. Calcd. For C_26_H_22_N_6_O_4_S (515): C, 60.69; H, 4.31; N, 16.33. Found: C, 60.44; H, 4.04; N, 16.09.

*N-(1H-Indazol-6-yl)-4-(2-phenylquinazolin-4-ylamino)benzenesulfonamide* (**17**). Yield, 89%; m.p. 149.9 °C. IR (KBr, cm^−1^): 3192, 3134 (NH), 3062 (CH arom.), 1635, 1624, 1600 (C=N), 1375, 1149 (SO_2_). ^1^H-NMR (DMSO-*d*_6_): 6.9–8.4 (m, 17H, Ar-H), 8.5, 10.1 (2s, 2H, SO_2_NH + NH, exchangeable with D_2_O), 12.9 (s, 1H, NH pyrazole, exchangeable with D_2_O). ^13^C-NMR (DMSO-*d*_6_): 100.5, 114.5, 114.8, 115.4 (2), 120.2, 121.3, 124.7, 126.3 (2), 127.0, 127.9, 128.2 (2), 128.6, 130.5 (2), 130.8, 131.8, 132.7, 133.2, 140.8, 144.0, 151.0, 158.3, 162.7. MS *m*/*z* (%): 493 (M^+^) (30.31), 117 (100). Anal. Calcd. For C_27_H_20_N_6_O_2_S (493): C, 65.84; H, 4.09; N, 17.06. Found: C, 65.46; H, 4.30; N, 17.29.

*4-(2-Phenylquinazolin-4-ylamino)-N-(quinoxalin2-yl)benzenesulfonamide* (**18**). Yield, 76%; m.p. 154.8 °C. IR (KBr, cm^−1^): 3194, 3134 (NH), 3059 (CH arom.), 1670, 1600 (C=N), 1338, 1161 (SO_2_).^1^H-NMR (DMSO-*d*_6_): 7.4–8.6 (m, 18H, Ar-H), 10.1 (s, 1H, SO_2_NH, exchangeable with D_2_O), 12.5 (s, 1H, NH, exchangeable with D_2_O). ^13^C-NMR (DMSO-*d*_6_): 114.5 (2), 114.6, 121.4, 123.5, 124.9, 126.3 (2), 126.5, 127.0, 127.7, 127.9, 128.2 (2), 128.7, 129.0, 129.2, 130.6, 130.9, 133.1, 134.0, 135.0, 138.4, 144.3, 146.6, 152.7, 162.7, 163.2. MS *m*/*z* (%): 505 (M^+^) (26.73), 175 (100). Anal.Calcd. For C_28_H_20_N_6_O_2_S (505): C, 66.65; H, 4.00; N, 16.66. Found: C, 66.93; H, 4.31; N, 16.34.

*4-Amino-N-(2-phenylquinazolin-4-yl)benzenesulfonamide* (**19**). A mixture of compound 1 (2.42 g, 0.01 mol), sulfanilamide (1.72 g, 0.01 mol) and anhydrous potassium carbonate (1.38 g, 0.01 mol) in dry dimethylformamide (15 mL) was refluxed for 18 h. The obtained solid was recrystallized from dioxane to give 19. Yield, 68%; m.p. 245.9 °C. IR (KBr, cm^−1^): 3394, 3209 (NH, NH_2_), 3059 (CH arom.), 1635, 1608 (C=N), 1375, 1134 (SO_2_). ^1^H-NMR (DMSO-*d*_6_): 7.0 (s, 2H, NH_2_, exchangeable with D_2_O), 7.5–9.0 (m, 13H, Ar-H], 11.9 [s, 1H, SO_2_NH, exchangeable with D_2_O]. ^13^C-NMR (DMSO-*d*_6_): 113.2 (2), 116.6, 124.4, 126.3 (2), 127.7, 127.8, 128.7 (2), 129.2, 129.8 (2), 131.7, 132.4, 132.8, 141.6, 157.7, 159.9, 162.7. MS *m*/*z* (%): 376 (M^+^) (22.25), 155 (100). Anal.Calcd. For C_20_H_16_N_4_O_2_S (376): C, 63.81; H, 4.28; N, 14.88. Found: C, 63.54; H, 4.01; N, 14.56.

*N-(4-(4-Aminophenylsulfonyl)phenyl)-2-phenylquinazolin-4-amine* (**20**). A mixture of 1 (2.42 g, 0.01 mol) and dapsone (2.48 g, 0.01 mol) in dry dimethylformamide (10 mL) was refluxed for 12 h. The obtained solid while hot was recrystallized from dioxane to give 20. Yield, 77%; m.p. 116.8 °C. IR (KBr, cm^−1^): 3415, 3375(NH, NH_2_), 3059 (CH arom.), 1618, 1595 (C=N), 1369, 1140 (SO_2_).^1^H-NMR (DMSO-*d*_6_): 5.9 (s, 2H, NH_2_, exchangeable with D_2_O), 6.5–8.6 (m, 17H, Ar-H), 10.1 (s, 1H, NH, exchangeable with D_2_O). ^13^C-NMR (DMSO-*d*_6_): 113.3 (2), 114.4 (2), 114.5, 122.9, 125.7 (2), 126.3, 127.0, 128.1 (2), 129.2 (4), 129.7, 130.2 (2), 131.8, 133.0, 143.8, 149.2, 152.7, 159.7, 162.7. MS *m*/*z* (%): 453 (M^+^) (32.53), 92 (100). Anal. Calcd. For C_26_H_20_N_4_O_2_S (453): C, 69.01; H, 4.45; N, 12.28. Found: C, 69.32; H, 4.71; N, 12.49.

*N,N′-(4,4′-Sulfonylbis(4,1-phenylene)bis(2-phenylquinazolin-4-amine)* (**21**). A mixture of 1 (4.84 g, 0.02 mol) and dapsone (2.48 g, 0.01 mol) in dry dimethylformamide (10 mL) was refluxed for 18 h. The obtained solid while hot was recrystallized from acetic acid to give 21. Yield, 68%; m.p. 197.9 °C. IR (KBr, cm^−1^): 3367, 3195 (NH), 3060 (CH arom.), 1622, 1597 (C=N), 1385, 1181 (SO_2_). ^1^H-NMR (DMSO-*d*_6_): 7.4–8.6 (m, 26H, Ar-H), 10.1 (s, 2H, 2NH, exchangeable with D_2_O). ^13^C-NMR (DMSO-*d*_6_): 113.4 (4), 114.5 (2), 126.3 (2), 126.8 (2), 127.9 (4), 128.2 (2), 128.6 (4), 129.7 (4), 131.8 (2), 133.1 (2), 134.0(2), 135.0 (2), 143.8 (2), 149.2 (2), 153.9 (2), 158.1 (2). MS *m*/*z* (%): 657 (M^+^) (9.30), 271(100). Anal. Calcd. For C_40_H_28_N_6_O_2_S (657): C, 73.15; H, 4.30; N, 12.80. Found: C, 73.37; H, 4.66; N, 13.09.

### 3.3. In-Vitro Anticancer Evaluation

#### 3.3.1. Cell Culture

Human cancer cell lines HeLa (cervical), A549 (lungs) and LoVo (colorectal) were grown in DMEM + GlutaMax (Invitrogen, Carlsbad, CA, USA), and MDA-MB-231 (breast) were grown in DMEM-F12 + GlutaMax) medium (Invitrogen), supplemented with 10% heat-inactivated bovine serum (Gibco) and penicillin-streptomycin (Gibco, Gaithersburg, MD, USA) at 37 °C in a humified chamber with 5% CO_2_ supply.

#### 3.3.2. Cytotoxicity Assay

The *in vitro* anticancer screening was done at Pharmacognosy Department, College of Pharmacy, King Saud University (Riyadh, Saudi Arabia). Cells were seeded (10^5^ cells/well) in 96-well flat-bottom plates (Becton-Dickinson Labware, Franklin Lakes, NJ, USA) a day before treatment and grown overnight. Compounds were dissolved in dimethyl sulfoxide (DMSO; Sigma) and finally prepared as 1.0 mg/mL stocks, respectively in the culture media. The final concentration of DMSO never exceeded 0.1% in the treatment doses. Six different doses of compounds (400, 200, 100, 50, 25 and 10 µM) were further prepared by diluting the stocks in culture media, and cells were treated (in triplicate/dose). Doxorubicin was included as standard reference drug (positive control) and untreated culture was considered as negative control. The cultures were further incubated for 48 hrs. At 48 h post-treatment, cell viability test was performed using TACS MTT Cell Proliferation and Viability Assay Kit (TACS) as per manufacturer’s instructions. The optical density (OD) was recorded at 570 nm in a microplate reader (ELx800, BioTek, Winooski, VT, USA) and cell survival fraction was determined. The cell survival fraction was calculated as [(A − B)/A], where A and B are the OD of untreated and of treated cells, respectively. The relation between surviving fraction and drug concentration is plotted to get the survival curve of each tumor cell line after the specified time. The concentration required for 50% inhibition of cell viability (IC_50_) was calculated and compared with the reference drug doxorubicin and the results are given in Table 1. Surviving curves for doxorubicin can be accessed as supplementary materials.

## 4. Conclusions

In this work, novel quinazoline-sulfonamide hybrids were synthesized and their *in vitro* anticancer activity was evaluated on four human cancer cell lines, among the tested compounds, two candidates (compounds **7** and **17**) showed effectiveness on the four cell lines, the active compounds could be considered as useful templates for further development to obtain more potent anticancer agent(s).

## Supplementary Materials

Supplementary materials can be accessed at: http://www.mdpi.com/1420-3049/21/2/189/s1.
