# Supplementary material for: Design, Synthesis and Anticancer Evaluation of Novel Quinazoline-Sulfonamide Hybrids
Source: Molecules. 2016 Feb 4;21(2):189. doi: 10.3390/molecules21020189 (PMC6274562; doi:10.3390/molecules21020189)
Supplement: Supplementary file 1 [file molecules-21-00189-s001.pdf]

Supplementary Materials: Design, Synthesis and Anticancer Evaluation of Novel Quinazoline-Sulfonamide Hybrids

Mostafa M. Ghorab, Mansour S. Alsaid, Mohammed S. Al-Dosari, Marwa G. El-Gazzar and Mohammad K. Parvez

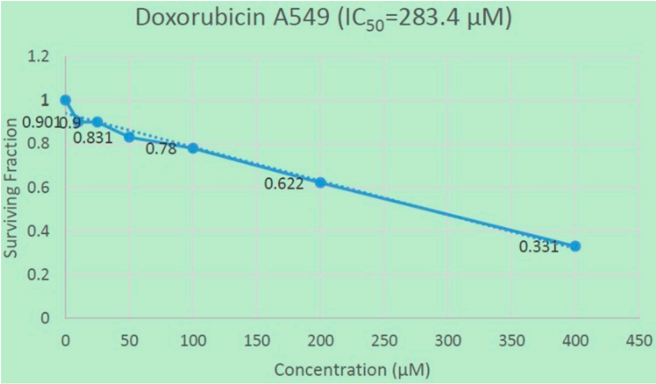

Figure S1. Surviving curve for doxorubicin on human lung cancer cell line (A549).

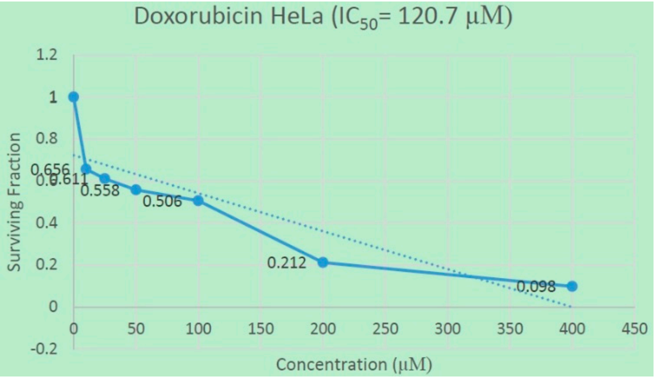

Figure S2. Surviving curve for doxorubicin on human cervical cancer cell line (HeLa).

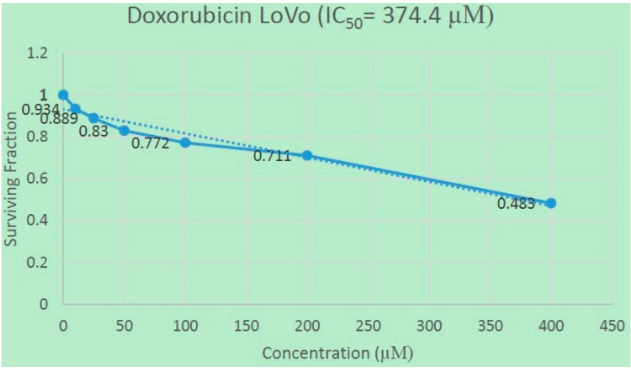

Figure S3. Surviving curve for doxorubicin on human colorectal cancer cell line (LoVo).

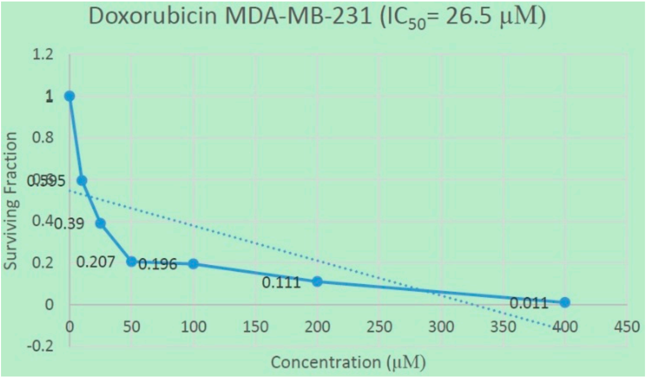

Figure S4. Surviving curve for doxorubicin on human breast cancer cell line (MDA-MB-231).
